# 

**Published:** 1888-05

**Authors:** 


WHOLE NUMBER CCCXXII

MAY, 1888.

NUMBER 10. VOLUME XXVII.

### THE BUFFALO Medical AND Surgical Journal

ESTABLISHED IN YEAR 1844.

LIBRARY INDEX S.G.O.

EDITORS:

THOS. LOTHROP, M. D. A. R. DAVIDSON, M. D.

ASSOCIATE EDITORS:

FLOYD S. CREGO, M. D. CARLTON C. FREDERICK, M. D.

FRED. R. CAMPBELL, M. D. FRANK H. POTTER, M. D.

EDWARD B. ANGELL, M. D., Rochester. N. Y.

Entered at the Post-Office at Buffalo, N. Y., as Second-class Mail Matter.

